# Antioxidant and Antimicrobial Effect of Biodegradable Films Containing Pitaya (*Stenocereus thurberi*) Extracts during the Refrigerated Storage of Fish

**DOI:** 10.3390/antiox12030544

**Published:** 2023-02-21

**Authors:** Daniela Castro-Enríquez, José M. Miranda, Marcos Trigo, Francisco Rodríguez-Félix, Santiago P. Aubourg, Jorge Barros-Velázquez

**Affiliations:** 1 Departamento de Investigación y Posgrado en Alimentos, University of Sonora, Hermosillo 83100, Sonora, Mexico; 2 Departamento de Química Analítica, Nutrición y Ciencia de los Alimentos, Facultad de Ciencias Veterinarias, Universidad de Santiago de Compostela, 27002 Lugo, Spain; 3 Departamento de Ciencia y Tecnología de Alimentos, Instituto de Investigaciones Marinas (CSIC), 36208 Vigo, Spain

**Keywords:** *Stenococcus thurberi*, polylactic acid, gelatin, fish muscle, refrigeration, lipid oxidation, lipid hydrolysis, microbial development, quality, natural preservatives

## Abstract

This study focused on the quality loss inhibition of fish muscle during refrigerated storage. Two parallel experiments were carried out that were focused on the employment of pitaya (*Stenocereus thurberi*) extracts in biodegradable packing films. On the one hand, a pitaya–gelatin film was employed for hake (*Merluccius merluccius*) muscle storage. On the other hand, a pitaya–polylactic acid (PLA) film was used for Atlantic mackerel (*Scomber scombrus*) muscle storage. In both experiments, fish-packing systems were stored at 4 °C for 8 days. Quality loss was determined by lipid damage and microbial activity development. The presence of the pitaya extract led to an inhibitory effect (*p* < 0.05) on peroxide, fluorescent compound, and free fatty acid (FFA) values in the gelatin–hake system and to a lower (*p* < 0.05) formation of thiobarbituric acid reactive substances, fluorescent compounds, and FFAs in the PLA–mackerel system. Additionally, the inclusion of pitaya extracts in the packing films slowed down (*p* < 0.05) the growth of aerobes, anaerobes, psychrotrophs, and proteolytic bacteria in the case of the pitaya–gelatin films and of aerobes, anaerobes, and proteolytic bacteria in the case of pitaya–PLA films. The current preservative effects are explained on the basis of the preservative compound presence (betalains and phenolic compounds) in the pitaya extracts.

## 1. Introduction

Marine species constitute a valuable source of healthy constituents, such as polyunsaturated fatty acids (PUFAs), high-quality proteins, lipophilic vitamins, and minerals [1]. For decades, refrigerated fish products have dominated the fish markets and represent a great proportion of human consumption [2]. However, the deterioration of sensory quality and nutritional value is likely to be produced in refrigerated fish as a result of different damage mechanisms, such as microbial activity and lipid oxidation [3]. Based on the current need for high-quality fresh products, refrigerated storage has been used together with other preservative strategies to increase the shelf life of the resulting seafood [4,5].

Among such complementary technologies, the development of packing films that incorporate sustainable ingredients and materials is considered a crucial strategy of food technology, with a view to extend the shelf-life time of food products in an environmentally friendly manner [6,7,8]. Thus, biodegradable and edible materials obtained from animals and plants, including polysaccharides, lipids, and peptides, have been reported to be profitable alternatives to synthetic packaging films [9,10]. Gelatin is a polysaccharide that can be obtained from diverse animal sources (porcine, bovine, and fish). It has been reported to constitute an effective biofilm to reduce quality loss of seafood and food in general [11,12]. Furthermore, the combined employment of gelatin packing films with different kinds of preservative compounds obtained from different natural sources, such as tea [13], macroalgae extracts [14,15], and microalgae protein concentrates [16], has successfully improved the retention of seafood quality. Polylactic acid (PLA) is a biodegradable thermoplastic polyester that has shown a preservative behavior during the refrigerated storage of fish species [17,18]. Its preservative effect has shown to be increased when complemented with different kinds of antioxidant (ascorbyl palmitate, α-tocopherol, macroalgae extracts, and sorbic acid) [19,20] and antimicrobial (nisin, potassium sorbate, and *Thymus foutanesil* essential oil) [21,22] compounds.

The genus *Stenocereus* includes plant species that grow in arid and semiarid climates from the southern United States to Central America [23]. Pitayas, a common name employed for *Stenocereus* fruits, are attracting great attention as a source of natural colorants and have been employed to increase the attractiveness to consumers of food in general [24,25,26]. Based on the high content of betalains and phenolic compounds in *Stenocereus* fruits, a growing interest has been addressed to the identification of these compounds and their bioactivity [27,28]. Betalains, water-soluble nitrogenous pigments, have been reported to present diverse biological activities, such as antioxidant, anti-inflammatory, and anticarcinogenic properties [29,30]. Meanwhile, phenolic compounds have been associated with biological activities based on phenolic hydroxyl groups and have shown remarkable preservative properties susceptible to application to seafood and food in general [31,32,33].

The objective of the present research was to inhibit the quality loss of fish muscle during refrigerated storage. Two parallel experiments were carried out based on the employment of a pitaya (*S. thurberi*) extract in biodegradable packing films. On the one hand, a pitaya–gelatin film was employed for hake (*Merluccius merluccius*) muscle storage. On the other hand, a pitaya–PLA film was used for Atlantic mackerel (*Scomber scombrus*) muscle storage. In both experiments, a control film without pitaya extract was also analyzed. All packing systems were stored at 4 °C for 8 days. The quality loss was determined by lipid damage (oxidation and hydrolysis) and microbial activity development.

## 2. Materials and Methods

### 2.1. Pitaya Extract and Biofilm System Preparation

Pitaya fruits (*S. thurberi*) were obtained in Carbó (State of Sonora, Mexico) in the June/July period of 2016. The fruits were washed, dried at 25 °C, placed in plastic bags, and stored under frozen conditions (−20 °C) for later use.

The pitaya extract was obtained following the methodology developed by Castro-Enríquez et al. [33]. Two grams of pitaya pulp without seeds and 34 mL of distilled water were mixed, sonicated for 27 min, and stirred for 20 min on a horizontal shaker in the dark. Subsequently, the mixture was centrifuged at 3500× *g* for 10 min. Then the supernatant was filtered through a Millipore membrane with a pore size of 1 kDa, at a pressure of 50 psi, with nitrogen gas. The extract obtained was lyophilized and stored in amber vials under frozen conditions (−20 °C) for later use.

Gelatin films were prepared from two separate solutions. For the first one, an aq. 10% (*w*/*v*) gelatin solution was prepared, heated at 60 °C, and stirred for 30 min. For the second solution, an aq. 1% (*w*/*v*) pitaya extract solution was prepared and stirred for 30 min. Subsequently, 5 mL of each solution was mixed, 0.238 mL of glycerol was added as a plasticizer, and the mixture was stirred for 30 min. After this time, the final solution was poured into molds that were 10 cm in diameter, and the molds were then placed in a convection oven at 50 °C for 3 h. Then the films were stored frozen at −20 °C for later use and labelled GEL-TR (gelatin treatment). Similarly, gelatin films were prepared without pitaya extract and labelled GEL-CT (gelatin control).

PLA pellets were ground in a blade mill (Thomas-Wiley, model 4, Swedesboro, NJ, USA). Subsequently, a 5% (*w*/*v*) solution was made in 10 mL of acetone and stirred for 30 min. After this time, 0.263 mL of polyethylene glycol diglycidyl ether plasticizer and 0.02 g of pitaya extract were added to the solution and stirred for 30 min. The solution was poured into molds that were 10 cm diameter and placed in a vacuum oven at 50 °C for 12 h for solvent evaporation. The films were stored frozen at −20 °C for later use and labelled PLA-TR (PLA treatment). Similarly, PLA films were prepared without pitaya extract and labelled PLA-CT (PLA control).

In both kinds of biofilms, the content of pitaya extract was chosen on the basis of previous trials carried out in our laboratory. Concentrations chosen did not modify the sensory and external properties of the fish muscle (i.e., odor and color).

### 2.2. Fish Material, Processing, and Sampling

Fresh hake (10 specimens; 350–375 g each) and fresh mackerel (12 specimens; 225–270 g each) were caught near the Galician Atlantic Coast (NW Spain), obtained at the Vigo (Spain) harbor, and transported to the laboratory. From catching till arrival at the laboratory, the fish were maintained on ice. The time elapsed for the transport from harbor to laboratory was 10 min. Specimens of both fish species were beheaded, eviscerated, skinned, filleted, and cut to obtain 45 muscle pieces of 65–75 g each. For each fish species, nine such pieces were divided into three groups (3 pieces per group) that were analyzed (lipid damage and microbial activity) independently and considered the initial substrate (*n* = 3).

For hake fish, the remaining 36 fish pieces of hake muscle were divided into 2 groups (18 pieces per group) and sealed/packed individually under the two abovementioned packing systems (GEL-CT and GEL-TR). Similarly, the remaining 36 fish pieces of mackerel muscle were divided into 2 groups (18 portions per group) and sealed/packed individually under the two abovementioned PLA-packing systems (PLA-CT and PLA-TR).

Packed muscle pieces were kept in a refrigerated room (4 °C) for an 8-day period, and sampling and analyses were performed on days 4 and 8. At each sampling time and for each fish species, 9 fish pieces were taken for analysis from each packing condition and divided into 3 groups (3 pieces for each group), which were studied independently (*n* = 3).

### 2.3. Lipid Damage Analyses

Lipids were extracted from the fish white muscle according to the method employed by Bligh and Dyer [34]. This method employs a single-phase solubilization of the lipids, using a chloroform–methanol (1:1) mixture. The results on lipid content were calculated as g lipid·kg^−1^ muscle.

The peroxide value (PV) was assessed spectrophotometrically (520 nm) (Beckman Coulter DU 640 spectrophotometer, Brea, CA, USA) in the lipid extract by following the method proposed by Chapman and McKay [35]. In this method, peroxides present in the lipid extract are reduced with ferric thiocyanate. The resulting values were calculated as meq. active oxygen·kg^−1^ lipids.

The thiobarbituric acid index (TBA-i) was determined according to the method proposed by Vyncke [36]. In it, the formation of thiobarbituric acid reactive substances (TBARS) by reaction between a trichloroacetic acid extract of the fish muscle and thiobarbituric acid was spectrophotometrically measured at 532 nm. The results were calculated as mg malondialdehyde·kg^−1^ muscle based on a standard curve prepared from 1,1,3,3-tetraethoxy-propane.

The fluorescent compound content (Fluorimeter LS 45; Perkin Elmer España; Tres Cantos, Madrid, Spain) was determined in the aqueous fraction, resulting from the lipid extraction of fish muscle [34]. According to previous research [37], fluorescence was measured at excitation/emission wavelengths of 393/463 and 327/415 nm. The relative fluorescence (RF) was calculated as follows: RF = F/F_st_, where *F* is the fluorescence measured at each excitation/emission wavelength pair and *F_st_* is the fluorescence intensity of a quinine sulphate solution (1 µg·mL^−1^ in 0.05 M H_2_SO_4_) at the corresponding wavelength pair. Results are given as the fluorescence ratio (FR), which was calculated as the ratio between the two RF values: FR = RF_393/463 nm_/RF_327/415 nm_.

The free fatty acid (FFA) content was determined on the lipid extract obtained from the fish muscle according to Lowry and Tinsley [38]. In this method, complex formation with cupric acetate–pyridine, followed by spectrophotometric (715 nm) assessment, is carried out. The results were calculated as g FFA·kg^−1^ muscle.

### 2.4. Microbial Activity Analyses

Portions of 10 g of fish muscle were dissected aseptically from refrigerated fish specimens, mixed with 90 mL of 0.1% peptone water (Merck, Darmstadt, Germany), and homogenized in sterilized stomacher bags (AES, Combourg, France), as previously described [39]. Serial dilutions from the microbial extracts were prepared in 0.1% peptone water in all cases.

Determination of total aerobes was carried out on plate count agar (PCA, Oxoid Ltd., London, UK) after incubation at 30 °C for 48 h. Assessment of anaerobes was carried out in the same manner, except that an anaerobic atmosphere kit was placed, together with the plates, inside the anaerobiosis jar. Determination of psychrotrophs was carried out on PCA after incubation at 7–8 °C for 7 days. *Enterobacteriaceae* were assessed in violet-red bile agar (VRBA) (Merck, Darmstadt, Germany) after incubation at 37 ± 0.5 °C for 24 h. Microorganisms exhibiting proteolytic or lipolytic phenotypes were determined in casein agar or tributyrin agar, respectively, after incubation at 30 °C for 48 h, according to previous research [40].

In all cases, bacterial counts were transformed into log CFU·g^−1^ muscle before undergoing statistical analysis. All analyses were conducted in triplicate.

### 2.5. Statistical Analysis

Results obtained from all chemical and microbiological determinations were subjected to ANOVA to explore differences resulting from the effect of the pitaya presence in the packing system and the refrigeration time. The comparison of average values was performed using the least-squares difference (LSD) method. In all cases, analyses were carried out using PASW Statistics 18 software for Windows (SPSS Inc., Chicago, IL, USA). In all cases, differences were considered significant for a confidence interval at the 95% level (*p* < 0.05).

## 3. Results and Discussion

### 3.1. Determination of Lipid Oxidation

A remarkable (*p* < 0.05) formation of peroxide compounds was detected in all kinds of fish samples with refrigeration time (Table 1). The presence of the pitaya extract in the gelatin-packing medium led to lower average values than in their counterpart control samples. Remarkably, differences were significant (*p* < 0.05) at day 4. In the case of mackerel samples, no inhibitory effect (*p* > 0.05) on peroxide formation was detected in the PLA–muscle system as a result of the presence of pitaya extract in the packing medium.

The average values obtained for TBARS formation showed a progressive increase with refrigeration time in all kinds of samples (Table 1). The presence of the pitaya extract led to lower average values in both hake and mackerel samples when compared to their corresponding control counterparts. Differences were found to be significant (*p* < 0.05) for PLA–mackerel samples but were not (*p* > 0.05) in the case of gelatin–hake samples.

The formation of fluorescent compounds revealed a substantial increase (*p* < 0.05) with storage time in all kinds of samples (Table 1). For both biopackaging systems, lower average FR values were detected in refrigerated fish that has pitaya extract in the packing media than in their counterpart controls. Differences were significant (*p* < 0.05) at day 8 in fish muscle in regard to both biopackaging systems.

Lipid oxidation is considered to be a complex damage mechanism since it involves the formation of a wide range of molecules, with most of them being unstable and, therefore, able to break down or interact with other molecules. This would be the case of primary (i.e., peroxides) and secondary (i.e., TBARS) lipid oxidation compounds, which are widely reported to break down and interact with nucleophilic-type molecules (proteins, peptides, free amino acids, aminated phospholipids, etc.) present in the fish muscle [15,41,42]. As a result of this interaction, the formation of fluorescent compounds has been reported to be produced, thus leading to a valuable way of assessing the advancement of the lipid oxidation mechanism development [43].

A wide range of previous studies revealed a high level of antioxidant properties in *Stenococcus* spp. juices [44,45]. This high antioxidant potential has been explained on the basis of the presence of substances with the capacity to donate an electron or a hydrogen atom to scavenge oxidizing compounds, such as ascorbic acid, betalains, and different phenolic compounds. Thus, Castro-Enríquez et al. [33] analyzed the antioxidant capacity (DPPH and ABTS assays) of *S. thurberi* and carried out the analytical determination of bioactive compounds. In such study, the UPLC–MS analysis revealed the presence of a wide range of phenolic (13–20 mg gallic acid equivalent·g^−1^) and betalain (0.94–1.37 mg·g^−1^) compounds. Among them, ferulic acid, catechin, caffeic acid, and quercetin could be mentioned. Remarkably, two phenolic compounds (i.e., gallic acid and resorcinol) were detected for the first time in species from the *Stenocereus* genus. Previously, total phenols (11.31 mg gallic acid equivalent·g^−1^ dry weight), total flavonoids (13.70 mg quercetin equivalent·g^−1^ dry weight), ascorbic acid (12.45 mg·100 g^−1^ fresh weight), and traces of carotenes were detected [27]. In addition, flavonol-type compounds such as quercetin, catechin, isorhamnetin, and kaempferol were detected in *S. thurberi*, *S. stellatus*, and *S. pruinosus* species [25,32].

Additionally, several in vitro and in vivo studies have reported the preservative and healthy properties of *Stenococcus* spp. juices. Thus, antioxidant (DPPH and FRAP assays) and antiproliferative (carcinogenic cell prevention) effects were detected in *S. pruinosus* juice that were explained by the presence of *p*-coumaric, gallic, caffeic, and vanillic acids [46]. Sandate-Flores et al. [47] showed the antioxidant properties (ABTS assay) of seed peptides obtained from *S. pruinosus* by sequential hydrolyzation of glutelin fractions with pepsin and trypsin–chymotrypsin. During an in vivo study, Ramírez-Rodríguez et al. [48] showed the antioxidant effect of *S. huastecorum* juice and explained it on the basis of the high content of betalains.

Concerning food applications, *Stenocereus* spp. fruits have been employed during the manufacture of several products, such as refreshing and alcoholic beverages, ice cream, and palettes [49,50]. However, to the best of our knowledge, no previous research has been reported taking advantage of the potential protecting properties for the preservation of seafood.

Previous research has reported the antioxidant effect derived from the inclusion of natural extracts in the biofilms used in the present research (gelatin and PLA). In general, this preservative effect has been justified as a result of the interaction between the antioxidant molecules present in the biofilm medium and the muscle of the marine species. Thus, García-Soto et al. [20] detected an inhibitory effect on lipid oxidation development (i.e., fluorescent compound formation) by including lyophilized *F. spiralis* and sorbic acid in a PLA packing film during the refrigerated storage (11 days at 4 °C) of megrim (*L. whiffiagonis*) fillets; this effect was explained on the basis of the preservative effect of the combination of sorbic acid and polyphenol compounds present in the alga. Additionally, the inclusion of *F. spiralis* protein concentrate in a gelatin-based film inhibited lipid oxidation development (TBARS and fluorescent compound formation) in refrigerated hake (*M. merluccius*) [33]. Recently, a lower fluorescent compound formation was observed in mackerel (*S. scombrus*) muscle as a result of employing a gelatin film containing lyophilized *F. spiralis* [15]. Concerning microalgae, the presence of a protein concentrate from *Spirulina platensis* in the gelatin packing film led to higher PUFA retention in refrigerated (4 °C) hake (*M. merluccius*) muscle [16]. A lower content of fluorescent compounds was obtained in mackerel (*S. scombrus*) muscle packed in a gelatin film containing a protein concentrate from *S. platensis* during storage at 4 °C [51].

### 3.2. Determination of Lipid Hydrolysis

**Figure 1:**
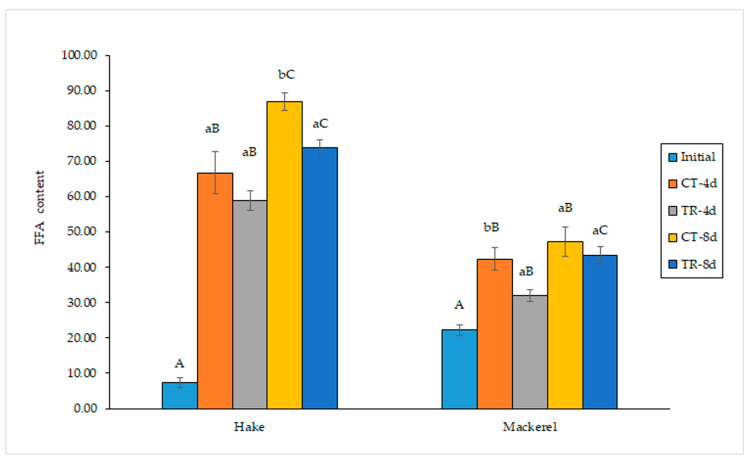
Determination of free fatty acid (FFA) content (g·kg^−1^ muscle) in refrigerated fish muscle stored under different packing conditions. Average values of three replicates (*n* = 3); standard deviations are indicated by bars. For each fish species and refrigeration time, values accompanied by different lowercase letters (a,b) indicate significant differences (*p* < 0.05) as a result of the pitaya extract’s presence in the packing medium. For each kind of fish sample, values accompanied by different capital letters (A–C) denote significant differences (*p* < 0.05) as a result of the storage time. Abbreviations of packing conditions: CT-4d and CT-8d (control samples refrigerated for 4 and 8 days, respectively), and TR-4d and TR-8d (pitaya-treated samples refrigerated for 4 and 8 days, respectively).

Increased (*p* < 0.05) lipid hydrolysis development was detected in all kinds of samples with storage time (Figure 1). The presence of the pitaya extract in both kinds of packing media (gelatin and PLA) led to lower average values at both storage times. For gelatin–hake samples, differences were significant (*p* < 0.05) at day 8, while significant differences (*p* < 0.05) were detected at day 4 for PLA–mackerel samples.

Lipid hydrolysis itself does not provoke nutritional losses. However, a direct relationship between FFA formation and loss of sensory acceptance (detrimental texture changes and development of off-odor and off-taste) has been reported [3,52]. Furthermore, FFA formation has shown a remarkable effect on lipid-oxidation development. This effect has been explained as a result of the lower oxidative stability of FFA compared to its corresponding phospholipids and triacylglycerols, since these molecules show a higher steric hindrance to oxidative reactions [37].

FFA formation in fish muscle during refrigerated storage has been explained as a result of endogenous and microbial enzyme activities [3]. The inhibitory effect observed in the current study can be explained on the basis of the antimicrobial properties of compounds present in the pitaya extract. This antimicrobial behavior is described and discussed in the following section.

No previous research has accounted for the effect of pitaya extracts on lipid hydrolysis development in seafood or food in general. However, previous research has reported the inhibitory effect on lipid hydrolysis development derived from the inclusion of different kinds of extracts obtained from natural resources in gelatin-derived films. This inhibitory effect on FFA content has been explained on the basis of the microbial activity inhibition. Thus, chitosan–gelatin packing inhibited the FFA formation in rainbow trout (*O. mykiss*) fillets [53] and Belanger’s croaker (*Johnius balangerii*) fillets [54] during refrigerated storage (4 ± 1 °C). An inhibitory effect of a crosslinked-gelatin film containing a spirulina (*S. platensis*) protein concentrate on FFA formation was detected in lean (hake, *M. merluccius*) [16] and fatty (mackerel, *S. scombrus*) [48] fish muscle. Likewise, the presence of a macroalga *F. spiralis* protein concentrate in gelatin-based packing led to a lower FFA content in hake (*M. merluccius*) muscle [14]. Recently, a lower lipid hydrolysis development was detected in mackerel (*S. scombrus*) muscle as a result of employing a gelatin film containing lyophilized *F. spiralis* [15].

### 3.3. Microbial Activity

**Figure 2:**
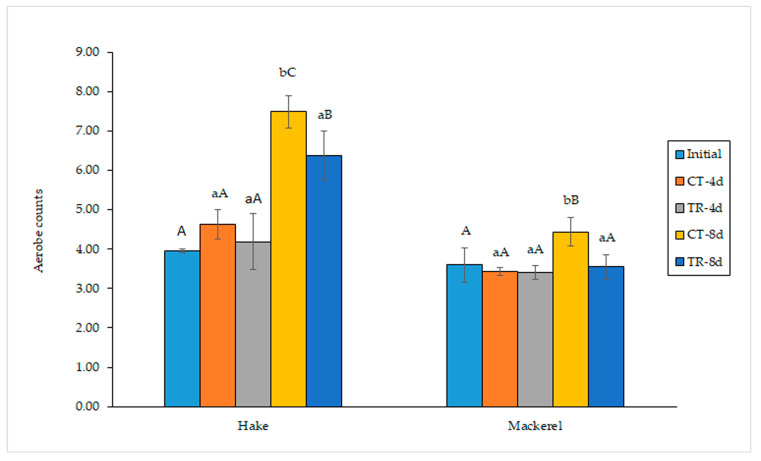
Aerobe development (log CFU·g^−1^ muscle) in refrigerated fish muscle stored under different packing conditions. Average values of three replicates (*n* = 3); standard deviations are indicated by bars. For each fish species and refrigeration time, values accompanied by different lowercase letters (a,b) indicate significant differences (*p* < 0.05) as a result of the pitaya extract’s presence in the packing medium. For each kind of fish sample, values accompanied by different capital letters (A–C) denote significant differences (*p* < 0.05) as a result of the storage time. Abbreviations of packing conditions are as expressed in Figure 1.

The investigation of aerobes in hake and mackerel muscle revealed a protective effect derived from the presence of the pitaya extract in the packaging films on both fish species (Figure 2). Thus, at advanced storage times (8 days), significant (*p* < 0.05) differences were observed between both hake batches, with the highest difference of 1.11 log units being observed on day 8, while on day 4, the differences were not significant (*p* > 0.05). Likewise, significant (*p* < 0.05) differences were observed between mackerel batches on day 8, with a difference of 0.89 log units being observed at that storage time, while on day 4, no significant (*p* > 0.05) differences were detected. Thus, in both fish species, a protective effect of the pitaya extract in the packing film on the microbial quality of fish was accomplished with regard to aerobe growth. Interestingly, this effect was observed for both types of fish species, despite their different lipid contents (i.e., 4.5–6.5 and 37.5–47.5 g·kg^−1^ muscle for hake and mackerel, respectively).

**Figure 3:**
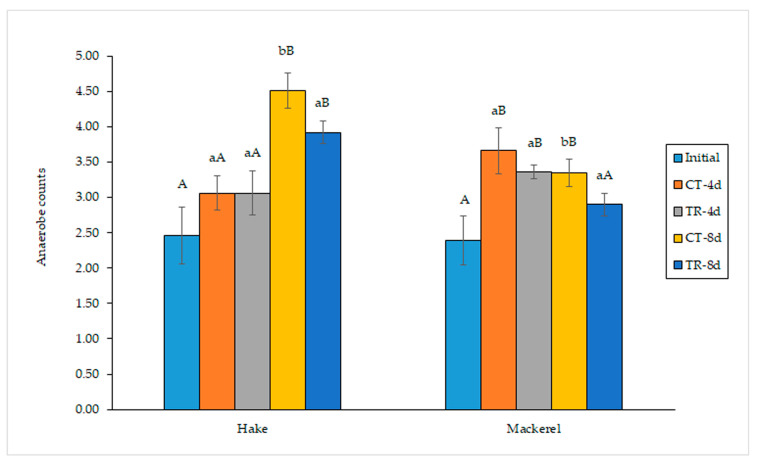
Anaerobe development (log CFU·g^−1^ muscle) in refrigerated fish muscle stored under different packing conditions. Average values of three replicates (*n* = 3); standard deviations are indicated by bars. For each fish species and refrigeration time, values accompanied by different lowercase letters (a,b) indicate significant differences (*p* < 0.05) as a result of the pitaya extract’s presence in the packing medium. For each kind of fish sample, values accompanied by different capital letters (A,B) denote significant differences (*p* < 0.05) as a result of the storage time. Abbreviations of packing conditions are as expressed in Figure 1.

With respect to the development of anaerobes in hake and mackerel muscle, the results are compiled in Figure 3. Likewise, as in the case of aerobes, the active film containing pitaya extract provided a protective effect on both fish species with regard to anaerobic growth. Thus, significant (*p* < 0.05) differences were observed between hake batches on day 8, with this difference rising to 0.59 log units. Likewise, significant (*p* < 0.05) differences were also observed between both mackerel batches at the end of storage time (day 8), with such differences increasing up to 0.45 log units. Thus, as in the case of aerobes, the presence of the pitaya extract in the packing film provided a significant (*p* < 0.05) reduction in aerobe growth in both fish species.

*Enterobacteriaceae* growth was also assessed, and the results for both fish species are compiled in Table 2. In this case, the results did not show any significant (*p* > 0.05) differences between batches in either of the two species tested. Remarkably, the negligible growth of *Enterobacteriaceae* observed throughout storage time (counts always below 1.75 log units) revealed the high initial quality of the fish specimens considered in this study.

Table 2 also compiles the results obtained regarding psychrotrophic growth in both fish species over storage time. Remarkably, different results were observed for each of the fish species tested with regard to the effect of the inclusion of the pitaya extract in the packing film. Thus, the presence of the pitaya extract in the films provided a significant (*p* < 0.05) protective effect on hake muscle at advanced storage times (day 8), with differences in the microbial counts higher than 1.0 log unit. In contrast, no significant (*p* > 0.05) differences between both mackerel batches were observed at any storage time, revealing a more limited protective effect of the active film on this fish species.

Specific spoilage organisms (SSOs), namely bacteria able to produce extracellular proteases and lipases, were also considered in this study, and the results are also compiled in Table 2. With respect to proteolytic bacteria, this microbial group contributes to the breakdown of myofibrillar proteins that provide the structure of fish muscle, thus negatively affecting fish quality [55]. In our study, the presence of the pitaya extract in the packing film significantly (*p* < 0.05) slowed the growth of proteolytic bacteria in hake muscle at advanced storage times (day 8). Remarkably, the differences between both hake groups on day 8 were 1.20 log units, a result that underlines a considerable reduction in the growth of this SSO group. In the case of mackerel, such significant (*p* < 0.05) differences were assessed at intermediate storage times (0.56 log units on day 4) but not on day 8.

In the case of lipolytic bacteria, this SSO group generates FFAs from triacylglycerols and phospholipids, with the subsequent development of fish rancidity, which is detrimental to fish quality [52,56]. In our study, a lower average development of lipolytic bacteria was observed in both hake and mackerel batches that had pitaya extract in their packing film as compared to their control counterpart batches. However, such differences were not significant (*p* > 0.05) at any storage time.

Pitaya extracts contain bioactive compounds such as polyphenols, alkaloids, flavonoids, and tannins, which are responsible for their antimicrobial activity [57]. Interestingly, this activity has been reported to be more intense against Gram-positives as compared to Gram-negatives. Such differences in their respective sensitivities seem to be related to the different cell wall composition of both bacterial groups. Thus, the presence of outer membrane and porins in Gram-negatives, but not in Gram-positives, has been related to less sensitivity to pitaya active components that, in contrast, would penetrate more easily into the inner membrane of Gram-positives and cause membrane disruption [58].

Previous research related to the antimicrobial effect of extracts derived from plants of the genus *Stenococcus* can be considered scarce and consists of in vitro studies. Thus, a methanolic extract of *S. pruinosus*, including alkaloids, triterpenes, and flavonoids, showed remarkable antifungal activity [59]. Later, Soto-Cabrera et al. [60] showed the in vitro antimicrobial activity of hexanic, ethyl acetate, and methanolic extracts of *S. stellatus* branches and explained it on the basis of the high polyphenol and flavonoid content. A recent study by Cheong et al. [58] reported the in vitro activity of pitaya extracts against several pathogenic and spoilage bacteria, such as *Bacillus cereus*, *Staphylococcus aureus*, *Enterococcus faecalis*, *Listeria monocytogenes*, *Escherichia coli*, *Proteus mirabilis*, *Proteus vulgaris*, *Pseudomonas aeruginosa*, *Salmonella typhi*, *Enterobacter cloacae*, *Enterobacter aerogenes*, *Yersinia enterocolitica*, and *Klebsiella pneumoniae.*

A wide number of previous works have reported the inhibitory effect on the microbial activity derived from the inclusion of bioactive compounds obtained from natural sources in gelatin and PLA films. In such studies, the antimicrobial effect was explained as a result of the interaction between the preservative molecules present in the biofilm medium and the muscle of the marine species. Thus, Min et al. [11] detected an inhibitory effect on Gram-negative pathogenic bacteria by employing a catfish-gelatin packing medium containing an origanum (*Thymus capitatus*) extract. An active packing strategy including lyophilized macroalga *F. spiralis* in a PLA film implied remarkable reductions of microbial growth in megrim (*Lepidorhombus whiffiagonis*) fillets under refrigeration conditions during an 11-day storage [20]. Remarkable reductions in aerobes, psychrotrophs, *Enterobacteriaceae*, proteolytic bacteria, lipolytic bacteria, and anaerobes were detected in refrigerated European hake (*M. merluccius*) that was packed in a gelatin-based film containing a *F. spiralis* protein concentrate [14]. A recent study also reported the inhibition of aerobes, psychrotrophs, and proteolytic bacteria in mackerel (*S. scombrus*) packed with an active gelatin film containing lyophilized *F. spiralis* [15]. Concerning microalga employment, *S. platensis* extracts, including polyhydroxybutyrate and phenolic compounds, allowed active packing strategies due to their antimicrobial activity when included in edible films [61]. Moreover, active films have also been prepared by including a protein concentrate from *S. platensis* in a gelatin film, leading to better microbial growth in refrigerated hake (*M. merluccius*) [16] and mackerel (*S. scombrus*) [51].

## 4. Conclusions

Two biodegradable films containing a pitaya (*S. thurberi*) extract were investigated as a novel packing strategy for the quality loss inhibition of fish muscle under refrigeration. As a result, the presence of the pitaya extract led to an inhibitory effect (*p* < 0.05) on the peroxide, fluorescent compound, and FFA values in the gelatin–pitaya system applied to hake muscle and on the formation of TBARS, fluorescent compounds, and FFAs in the PLA–pitaya system applied to mackerel muscle. Concerning microbial development, the inclusion of the pitaya extract in the packing films slowed down (*p* < 0.05) the growth of aerobes, anaerobes, psychrotrophs, and proteolytic bacteria in the case of the pitaya–gelatin film and of aerobes, anaerobes, and proteolytic bacteria in the case of pitaya–PLA film. Based on the results obtained, an antioxidant and antimicrobial effect is concluded for the pitaya extract in both packing systems. The preservative effect is justified by the presence of different kinds of preserving molecules, such as betalains and phenolic compounds, in the pitaya extract. Such molecules would interact with the fish muscle and lead to an inhibitory effect on lipid oxidation and microbial activity.

The proposed preservation strategy agrees with the present efforts in the search for effective protective compounds (antioxidant and antimicrobial) obtained from natural sources to replace synthetic preservatives in food in general. Additionally, the use of biodegradable films constitutes a promising basis to reduce waste-material production during the different steps of fish-product commercialization. The current study constitutes a novel and beneficial strategy to enhance the quality of commercial fresh fish and facilitate environmental sustainability and the circular economy. Further research focused on the optimization of pitaya extract addition to the biofilms tested should be considered to apply this active packing strategy. This optimization should take into account the physicochemical properties of the resulting films in order to provide a safe, nutritious, and attractive fresh product to the consumer. Furthermore, maintenance of the sensory and external properties of the fish muscle ought to be guaranteed.

## Figures and Tables

**Table 1:** Determination of lipid oxidation * in refrigerated fish muscle stored under different packing conditions **.

Quality Index	Refrigerated Time (Days)	Hake Muscle		Mackerel Muscle	
		**Packing Condition**		**Packing Condition**	
		**GEL-CT**	**GEL-TR**	**PLA-CT**	**PLA-TR**
PV (meq. active oxygen·kg^−1^ lipids)	Initial	0.72 ± 0.20 A	0.72 ± 0.20 A	0.36 ± 0.05 A	0.36 ± 0.05 A
	4	3.20 ± 0.12 bB	2.89 ± 0.07 aB	5.49 ± 0.69 aB	5.86 ± 1.74 aB
	8	6.96 ± 0.49 aC	6.20 ± 0.41 aC	12.59 ± 0.43 aC	11.23 ± 2.18 aC
TBA-i (mg malondialdehyde·kg^−1^ muscle)	Initial	0.32 ± 0.17 A	0.32 ± 0.17 A	0.71 ± 0.42 A	0.71 ± 0.42 A
	4	0.66 ± 0.10 aB	0.49 ± 0.17 aA	4.84 ± 0.33 bB	3.34 ± 0.45 aB
	8	0.83 ± 0.18 aB	0.64 ± 0.18 aA	5.09 ± 0.12 bB	4.60 ± 0.17 aC
Fluorescence ratio	Initial	0.76 ± 0.17 A	0.76 ± 0.17 A	2.51 ± 0.70 A	2.51 ± 0.70 A
	4	2.15 ± 0.16 aB	1.98 ± 0.32 aB	4.39 ± 0.48 aB	3.63 ± 0.50 aA
	8	4.47 ± 0.23 bC	3.58 ± 0.31 aC	8.29 ± 1.15 bC	5.26 ± 0.41 aB

* Average values of three replicates (*n* = 3) ± standard deviations. For each fish species and refrigeration time, values accompanied by different lowercase letters (a,b) indicate significant differences (*p* < 0.05) as a result of the pitaya extract’s presence in the packing medium. For each kind of fish sample, values accompanied by different capital letters (A–C) indicate significant differences (*p* < 0.05) as a result of the storage time. ** Packing conditions: GEL-CT (hake muscle packed in gelatin biofilm; gelatin control), GEL-TR (hake muscle packed in gelatin biofilm containing pitaya extract), PLA-CT (mackerel muscle packed in polylactic acid biofilm; polylactic acid control), and PLA-TR (mackerel muscle packed in polylactic acid biofilm containing pitaya extract).

**Table 2:** Microbial (*Enterobacteriaceae*, psychrotrophs, proteolytic bacteria, and lipolytic bacteria) development (log CFU·g^−1^ muscle) * in refrigerated fish muscle stored under different packing conditions **.

Microbial Group	Refrigerated Time (Days)	Hake Muscle		Mackerel Muscle	
		**Packing Condition**		**Packing Condition**	
		**GEL-CT**	**GEL-TR**	**PLA-CT**	**PLA-TR**
*Enterobacteriaceae*	Initial	1.0 ± 0.0 A	1.0 ± 0.0 A	1.0 ± 0.0 A	1.0 ± 0.0 A
	4	1.74 ± 0.52 aB	1.36 ± 0.39 aA	1.0 ± 0.0 aA	1.0 ± 0.0 aA
	8	1.10 ± 0.17 aAB	1.16 ± 0.27 aA	1.0 ± 0.0 aA	1.0 ± 0.0 aA
Psychrotrophs	Initial	3.79 ± 0.48 A	3.79 ± 0.48 A	2.75 ± 0.26 A	2.75 ± 0.26 A
	4	4.91 ± 0.76 aA	4.87 ± 0.65 aA	3.56 ± 0.41 aB	3.75 ± 0.32 aB
	8	7.40 ± 0.22 bB	6.37 ± 0.32 aB	4.46 ± 0.48 aB	4.35 ± 0.60 aB
Proteolytic bacteria	Initial	2.74 ± 0.67 A	2.74 ± 0.67 A	2.43 ± 0.38 A	2.43 ± 0.38 A
	4	4.92 ± 0.36 aB	5.15 ± 0.59 aB	4.44 ± 0.32 bB	3.88 ± 0.61 aB
	8	7.28 ± 0.57 bC	6.08 ± 0.36 aB	4.84 ± 0.47 aB	4.97 ± 0.61 aB
Lipolytic bacteria	Initial	2.00 ± 0.00 A	2.00 ± 0.00 A	2.00 ± 0.00 A	2.00 ± 0.00 A
	4	3.69 ± 0.64 aB	3.33 ± 0.92 aB	3.21 ± 0.41 aB	3.33 ± 0.73 aB
	8	4.05 ± 0.95 aB	3.27 ± 0.89 aB	3.98 ± 0.28 aC	3.53 ± 0.92 aB

* Average values of three replicates (*n* = 3) ± standard deviations. For each fish species and refrigeration time, values accompanied by different lowercase letters (a,b) indicate significant differences (*p* < 0.05) as a result of the pitaya extract’s presence in the packing medium. For each kind of fish sample, values accompanied by different capital letters (A–C) indicate significant differences (*p* < 0.05) as a result of the storage time. ** Abbreviations of packing conditions are as expressed in Table 1.

## Data Availability

Not applicable.
